# The evolutionary game of establishing a remote consultation system based on the downward allocation of medical resources in a medical alliance

**DOI:** 10.1371/journal.pone.0305747

**Published:** 2024-07-18

**Authors:** Tao Du, Jinyu Li, Liuyuanyuan Guo, Xiaohu Wang, Qiuyue Zhu

**Affiliations:** School of Economics and Management, Yanan University, Yanan, Shaanxi, China; Zhejiang Gongshang University, CHINA

## Abstract

As a crucial component of hierarchical diagnosis and treatment systems, medical alliances in China are responsible for promoting the downward allocation of high-quality medical resources. Remote consultation, as an essential means to achieve this goal, is of practical importance in the realization of resource sharing between hospitals within medical alliances in China. The existing research on the construction of remote consultations within medical alliances has achieved fruitful results in both theory and practice. However, the establishment of remote consultation involves many factors, and the current research mainly focuses on the influence of traditional economic profit and loss on the construction of remote consultation. In view of the practical problems existing in the operation of medical and health services in China, such as the need to improve the capacity of primary medical and health services and the poor sinking effect of high-quality medical resources, it is of great importance to systematically study the promotion strategy of the construction of remote consultation within the medical alliance to build a reasonable order of medical treatment. Therefore, by determining the logical path formed by the remote consultation channel and on the basis of traditional profit and loss parameters, this paper fully considers the relevant influence of the resource sinking utility caused by the remote consultation channel. The stability of the evolutionary system is analyzed, and a numerical simulation is used to explore the impact of key parameters on system evolution. The research results indicate that the establishment of a remote consultation system between hospitals at different levels is primarily influenced by factors such as the initial proportion of the establishment strategy chosen by both parties, the establishment cost, the distribution proportion of the government subsidy, the distribution proportion of the economic benefit, and the effectiveness proportion in the utility derived from the downward allocation of resources and reputational damage. The findings suggest that moderate to high levels of reputation loss do not significantly influence the final decision-making process for either party. Government subsidies can have an impact on hospital decision-making in the early stages, and in the long term, the resource sinking utility is more appealing than the economic benefits. To a certain extent, this study enriches the related research on remote consultation and the sinking of high-quality medical resources, provides reliable theoretical and method support for the sinking of high-quality medical resources, promotes the construction of remote consultation in medical alliances in China, and provides a decision-making reference and basis for the government and health administrative departments to formulate relevant policies.

## Introduction

A medical alliance in China is a health care organization formed by the vertical or horizontal integration of resources of different levels and categories of medical institutions within a certain region in China based on the principle of government-led planning and coordination [1]. The four primary models of medical alliances in China include urban medical groups, county medical communities, regional specialty alliances, and remote medical collaboration networks. As a crucial approach to implementing hierarchical diagnosis and treatment systems, medical alliances in China aim to integrate medical resources and promote the downward allocation of high-quality medical resources, which can effectively improve the capacity for primary health care services within medical alliances and increase two-way referral services [2]. Remote consultation refers to the use of audio, video, and electronic information technology to provide health care services or assistance to remote areas with limited access to medical resources [3], which achieves the downward allocation of high-quality resources and improves patient treatment outcomes. In 2020, due to the lockdown in Wuhan caused by the COVID-19 pandemic, experts at the provincial and municipal levels were limited by geographical distances and resorted exclusively to online consultations to discuss cases to improve treatment plans [4]. Remote consultation has played an important role in the prevention, control, and treatment of global epidemics due to its spatially unrestricted nature [5–7]. Research on remote consultation has yielded abundant findings both domestically and internationally, with a primary focus on two aspects. One is the investigation of remote consultation itself, including its status, application, and the willingness and behavior of relevant subjects to adopt it [8–10]. The other aspect is to regard remote consultation as one way to integrate health care resources within the system [11] by leveraging internet technology to promote the flow of high-quality medical resources, medical information, and services across regions to improve the uneven distribution of medical resources [12–15]. Remote consultation is an effective approach for facilitating the downward allocation of high-quality medical resources [12–16], enabling primary health care institutions to quickly improve their medical service capabilities according to the principle of ‘seeking local medical treatment and reducing mobility’. This approach is of practical importance for achieving the downward allocation of high-quality medical resources.

The downward allocation of medical resources refers to the redistribution of high-quality medical resources from tertiary hospitals to primary health care institutions within the medical system [17]. An effective approach is to address the disparity in service capacity among different medical facilities and improve the equity of health care services for residents [18]. Current research on the downward allocation of medical resources focuses primarily on the ‘how’ and ‘what to do’ aspects. The former assesses the fairness, equality, and efficiency of medical resource allocation; the latter is the solution to the existing problems of China’s downward allocation of medical resources. Judging from the operation of China’s hierarchical diagnosis and treatment service system, the challenge of promoting high-quality medical resources to underserved areas persists due to conflicting interests among medical institutions [19]. Therefore, there are varying degrees of quantity and quality imbalance in the medical and health resources across all levels of health care facilities. When the imbalance in the allocation of medical and health resources is greater and the force of downward allocation is smaller, the efficiency of utilizing medical and health resources is lower [20–22]. This situation has prompted academia and practical research fields to constantly seek effective ways to alleviate it. The ‘what to do’ is the proposed solution for the existing problems of the downward allocation of medical resources in China. Many policies, programs, and methodologies have been proposed to facilitate the downward allocation of medical resources. These measures include establishing medical alliances in China [23], implementing a hierarchical diagnosis and treatment system [24], expanding the supply of medical resources to community hospitals through remote health care services [25], and enhancing the stockpile of medical and health resources at the primary level. A small number of studies have focused on determining the downward allocation rate of high-quality medical resources in hierarchical diagnosis and treatment systems [15]. They have used game models to find the optimal rate of the downward allocation of tertiary hospitals to primary hospitals, thus improving medical accessibility and social benefits [17, 18]. It is evident that the downward allocation of resources is not only an effective way to optimize the allocation of existing medical resources but also an important means to enhance the service capabilities of primary medical and health care in China.

In essence, in the promotion of remote consultation in a medical alliances in China, each subject, on the basis of maximizing his or her own benefits, repeatedly adjusts his or her own strategic choices by referring to the decisions of other subjects and finally makes most subjects converge to a stable state that satisfies the maximum interests of all parties through interaction, coordination, and cooperation among the subjects. This is consistent with the adjustments that evolutionary games [26] need to make to reach the ‘optimal solution’ by constructing a dynamic learning model and then judging evolutionary stability points with the help of replicated dynamic equations. At present, the evolutionary game method has been widely applied to medical and health research. In the selection of game subjects, government departments, hospitals, doctors, and patients are considered the main representatives [27–30]. In terms of parameter setting, most studies focus on parameters related not only to costs and profit and loss, such as the cost of various facilities and labor costs for establishing remote consultation, but also to the benefits gained after the establishment and the losses in the absence of establishment [27–31]. Although the current research is relatively complete, it is more reasonable to construct an evolutionary game model that fully considers the parameters related to the sinking of high-quality medical resources in view of the realistic difficulties of the unbalanced medical order, weak primary medical service ability, and inaccurate sinking of high-quality medical resources in China.

**Fig 1:**
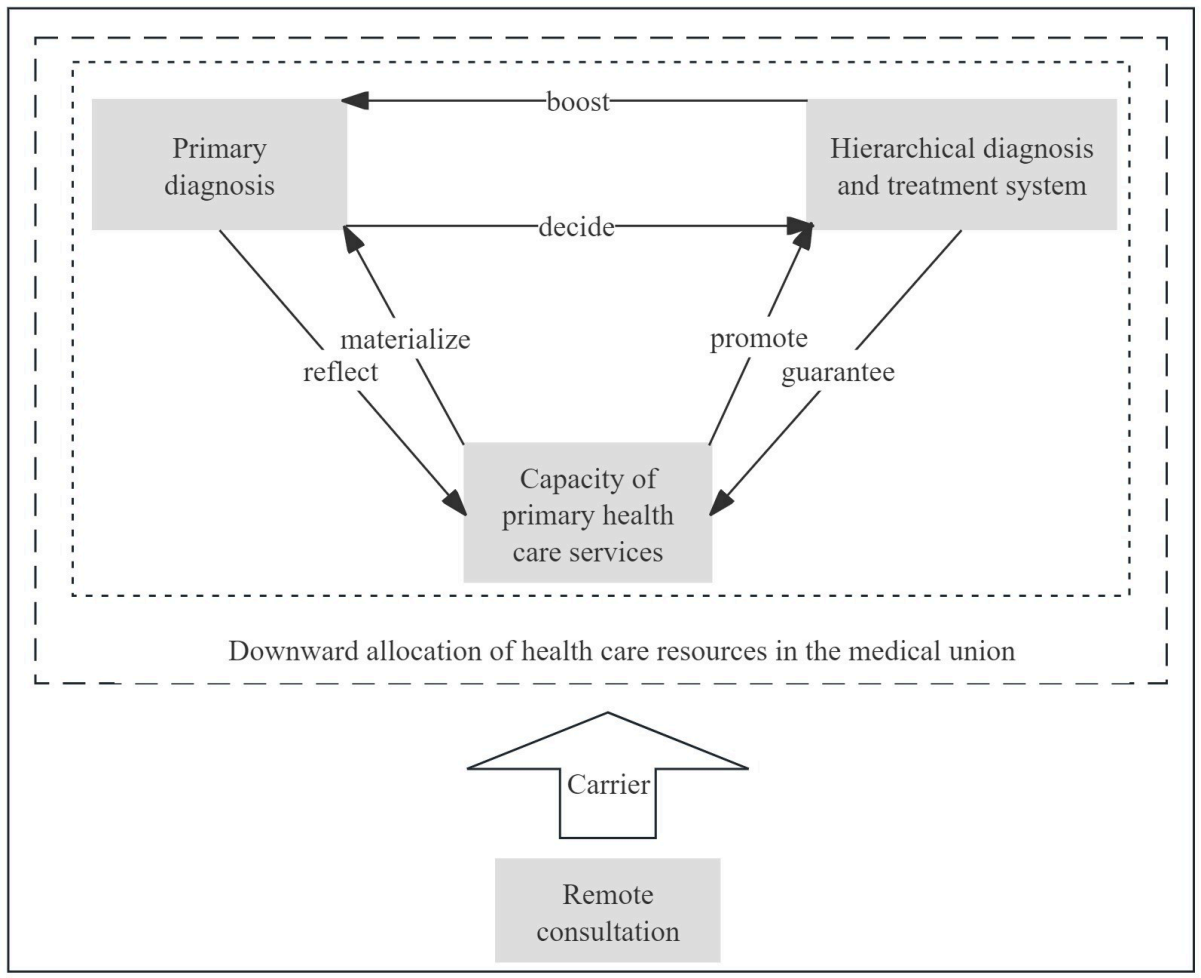
There was a logical relationship among the primary diagnosis, hierarchical diagnosis and treatment system, and remote consultation.

Accordingly, this study focuses on the question of how to expand the increase in medical supply to primary health care institutions. As shown in Fig 1, the implementation of a hierarchical diagnosis and treatment system is crucial for optimizing medical management and promoting structural reform on the supply side of health care services. The high rate of initial diagnosis at the primary care level provides compelling evidence for the rational allocation of medical resources and improved accessibility to health care services. The enhancement of primary health care services reflects the equalization of fundamental health and medical services. The primary diagnosis, hierarchical diagnosis and treatment system, and remote consultation are interconnected and mutually reinforced [32], creating a pathway for the downward allocation of medical resources within medical alliances in China. Remote consultation serves as a crucial carrier that ensures the smooth operation of this pathway. Therefore, remote consultation has significant practical implications for facilitating the downward allocation of medical resources. This study investigates the strategic interactions between tertiary hospitals (THs) and primary hospitals (PHs) in medical alliances with respect to the decision to implement a remote consultation system from the perspective of the downward allocation of resources. A two-party evolutionary game model is constructed to explore the expected benefits of THs and PHs under different choices to establish remote consultations. This is followed by the construction of a replicator dynamic equation to analyze the equilibrium state and evolution process of the game. To a certain extent, this study enriches the related research on remote consultation and the sinking of high-quality medical resources, provides reliable theoretical and method support for the sinking of high-quality medical resources, promotes the construction of remote consultation in medical alliances in China, and provides a decision-making reference and basis for the government and health administrative departments to formulate relevant policies.

## Problem description, symbol explanation, and model establishment

### Description of the problem

**Fig 2:**
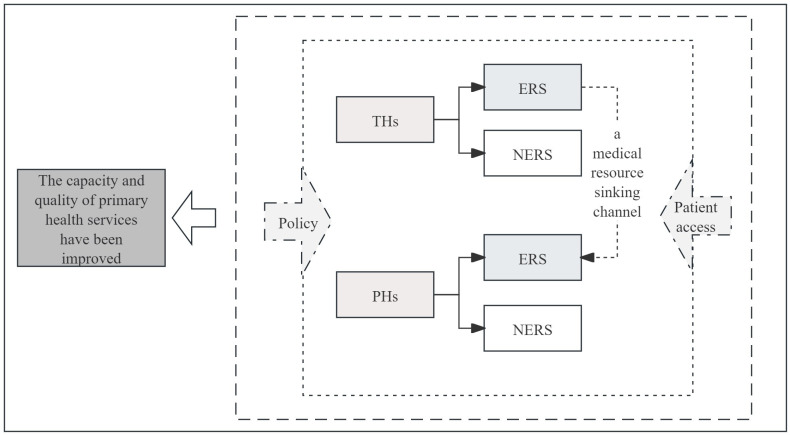
The downward allocation of medical resources can improve the capacity and quality of primary health services.

Promoting the downward allocation of medical resources and improving primary health care service capacity has consistently been a focal point in China’s health policy reform. However, the predicament of the downward allocation of medical resources persists within the medical alliance system in China [19], and the key to resolving this problem lies in establishing an efficient channel for the allocation of medical resources, with remote consultation as its carrier. As shown in Fig 2, in a medical alliance system in China, there are two groups: THs and PHs. To realize the sinking of high-quality medical resources in medical alliances, it is necessary to build a remote consultation channel. The formation of this channel means that both sides will build a remote consultation system, thus promoting the flow and allocation of medical resources in medical alliances in China. PHs and THs have two different options: either ‘establish a remote consultation system (ERS)’ or ‘do not establish one (NERS)’. However, if both sides choose NERS or if one side chooses ERS while the other chooses NERS, then the remote consultation channel is blocked, and medical resources still cannot sink. Therefore, the establishment of a remote consultation system by both THs and PHs is essential for the formation of a downward allocation of medical resources channel based on remote consultations, which would allow the effective sharing and utilization of resources between different levels of hospitals in medical alliances in China. This can lead to an improvement in both the capacity and quality of primary health care services.

To address the aforementioned issues and provide a more comprehensive characterization of the game process between THs and PHs, we make the following assumptions.

Assumption 1: According to General Office of the State Council Issuing the “Guiding Opinions on Promoting the Construction and Development of Medical Consortiums. The evolutionary game is between the group of THs and PHs within the medical alliance in China, which involves multiple hospital entities. Both THs and PHs participate in remote consultations.

Assumption 2: Both parties have different strategic choices [28]. THs can choose to establish a remote consultation system with a strategy set of ERS, NERS, and the probability of choosing ERS is denoted by *x*(*x* ∈ [0, 1]), while the probability of choosing NERS is represented by 1 − *x*. Similarly, PHs have a strategy set of ERS, NERS, and the probability of choosing ERS is *y*(*y* ∈ [0, 1]), whereas the probability of selecting NERS is 1 − *y*.

Assumption 3: There is asymmetric information between the parties regarding the establishment of a remote consultation system, and both parties possess limited rationality [29]. That is, at the beginning of the evolutionary game, neither party can make the optimal strategic choice, and they need to continuously adjust their own strategic choices based on each other’s actions to achieve an equilibrium state in the evolutionary game.

### Symbol description

#### Aspects of costs and subsidies

The construction of a remote consultation channel within a medical alliance primarily involves the establishment of a remote consultation system between THs and PHs in China. The components of the remote consultation system mainly include hardware equipment and software systems. The costs mainly consist of equipment investment and depreciation, promotional and publicity expenses, training expenses, management expenses, salaries (for doctors and full-time staff), and system maintenance expenses [33], and the investment of THs usually exceeds that of PHs. Simultaneously, to facilitate the reform of the supply side of medical and health services and improve the service capabilities of PHs within medical alliances in China, the government actively promotes the establishment of remote consultation and provides differential subsidies to hospitals at various levels. Notice on the Special Application for the Construction of Primary-level Telemedicine Rooms within the Provincial Budget for 2020 of the Hunan Provincial Development and Reform Commission can be used as evidence. Therefore, the present article assumes that the respective costs of establishing remote consultation between THs and PHs are *C*_1_/*C*_2_. The government has allocated a total subsidy *S* for the establishment of remote consultation systems in both types of hospitals, with the proportion *α*(*α* > 0) designated for THs and the proportion 1 − *α* allocated for PHs.

#### Aspects of benefits

The establishment of a remote consultation system between THs and PHs offers both economic and social benefits [34, 35]. The social benefits for PHs include providing better medical services and helping patients see a doctor at the primary level. The social benefits for THs include more time and energy to treat difficult serious diseases in the region and to make positive contributions to improving the medical service level in China by improving their own treatment level. Both parties bring different costs and benefits to one another when deciding whether to establish a remote consultation system. The implementation of a benefit-sharing mechanism within alliances can improve patient satisfaction and facilitate the coordination of the provision of medical services [34, 36]. Therefore, this article assumes that the economic benefits obtained jointly by the THs and PHs for establishing a remote consultation system are *R*, with the proportion allocated to the THs being *β*(*β* > 0) and the proportion allocated to the PHs being 1 − *β*. *W*_1_/*W*_2_ are the social benefits generated by the establishment of remote consultation systems by both parties.

#### Aspects of the utility of the downward allocation of resources

The establishment and efficient operation of remote consultation channels can facilitate the effective downward allocation of high-quality medical resources within medical alliances in China. On the one hand, they can enhance not only the ability to provide services but also the quality of PHs. On the other hand, as the service capabilities of PHs improve, patients gradually prefer them as their primary choice for medical treatment. To some extent, this can alleviate the situation where high-quality medical resources are occupied by THs [37, 38]. However, when remote consultation channels are not available, the service capabilities and quality of PHs do not meet the medical needs of patients who elect to obtain treatment there. Furthermore, when patients choose to seek treatment at THs, they are also easily affected by the problem of overcrowding [38]. The sinking effects of medical resources on PHs and THs after remote consultation are smooth, including the brand effect caused by high patient satisfaction, distance education and training, and medical resource sharing and scheduling, and can reduce the number of outpatient services for common patients in THs, release the pressure of scarce medical resources, and realize the benefits of scientific research, teaching, and management decision-making in medical alliances in China. In contrast, patients have doubts about the service capabilities and treatment efficiency of both THs and PHs, which leads to complaints, reputational damage, and a negative impact on both types of hospitals. Therefore, we assume that both THs and PHs can achieve resource sinking utility *E* when the remote consultation channel operates smoothly, where *ϕ*(*ϕ* > 0) and 1 − *ϕ* represent the proportions of THs and PHs, respectively, while *Q*_1_/*Q*_2_ represent the reputation damage and negative impact caused by complaints and improper performance of THs and PHs, respectively, in the case of disruptions in the remote consultation channel. The parameter settings for this study are generally presented in Table 1.

**Table 1:** Parameter symbols and meanings.

Parameter Symbols	Meanings
*C*_1_	The expenses associated with the establishment of a remote consultation system for THs
*C*_2_	The expenses associated with the establishment of a remote consultation system for PHs
*S*	Government financial subsidy for THs and PHs to establish remote consultation system
*R*	The economic benefits jointly acquired by THs and PHs subsequent to the establishment of a remote consultation system
*W*_1_	The establishment of a remote consultation system generates social benefits for THs
*W*_2_	The establishment of a remote consultation system generates social benefits for PHs
*E*	Both THs and PHs chose the ERS strategy to obtain the sinking utility of medical resources
*Q*_1_	Reputation damage and negative impact arise from complaints and improper performance when remote consultation channels are not seamless and THs fail to establish an effective remote consultation system
*Q*_2_	Reputation damage and negative impact arise from complaints and improper performance when remote consultation channels are not seamless and PHs fail to establish an effective remote consultation system
*α*	Allocation ratio pertaining to government subsidies
*β*	Allocation ratio pertaining to economic benefits
*ϕ*	Proportion of utility derived from the downward allocation of resources

### Model establishment

Based on the aforementioned assumptions, we construct a two-player evolutionary game model that incorporates bounded rationality. The payoff matrix for both THs and PHs is presented in Table 2.

**Table 2:** Revenue matrix for THs and PHs.

PHs			
		ERS(*y*)	NERS(1 − *y*)
THs	ERS(*x*)	−*C*_1_ + *αS* + *W*_1_ + *ϕE*	−*C*_1_ + *αS* + *W*_1_ − *Q*_1_
		−*C*_2_ + (1 − *α*)*S* + (1 − *β*)*R* + *W*_2_ + (1 − *ϕ*)*E*	−*Q*_2_
	NERS(1 − *x*)	−*Q*_1_	−*Q*_1_
		−*C*_2_ + (1 − *α*)*S* + *W*_2_ − *Q*_2_	−*Q*_2_

## Analysis of the evolutionary game model of remote consultation promoted by the downward allocation of medical resources

### Replicator dynamic equation for the evolutionary game of THs and PHs within a medical alliance

According to the aforementioned assumptions, let *U*_11_ and *U*_12_ represent the payoffs for THs choosing the ERS strategy and the NERS strategy, respectively:

$$\begin{array}{c} \begin{array}{cc} U_{11} & =y\left(-C_{1}+\alpha S+\beta R+W_{1}+\phi E\right)+\left(1-y\right)\left(-C_{1}+\alpha S+\beta R+W_{1}-Q_{1}\right)\\ & =y\left(\phi E+\beta R+Q_{1}\right)-C_{1}+\alpha S+W_{1}-Q_{1} \end{array} \end{array}$$ (1)

$$\begin{array}{c} U_{12}=-yQ_{1}-\left(1-y\right)Q_{1}=-Q_{1} \end{array}$$ (2)

The mean payoff for THs would be the following:

$$\begin{array}{c} {\bar{U}}_{1}=xU_{11}+\left(1-x\right)U_{12} \end{array}$$ (3)

A replicator dynamic equation is a type of differential equation that characterizes the temporal evolution of strategy frequencies or distributions within a population. Therefore, the replicator dynamic equation for THs choosing the ERS strategy can be expressed as follows.

$$\begin{array}{c} \begin{array}{cc} F(x) & =\frac{dx}{dt}=x\left(U_{11}-{\bar{U}}_{1}\right)=x\left(1-x\right)\left(U_{11}-U_{12}\right)\\ & =x\left(1-x\right)[y\left(\phi E+\beta R+Q\right)-C_{1}+\alpha S+W_{1}] \end{array} \end{array}$$ (4)

Similarly, the rewards for PHs who select the ERS and NERS strategies are denoted as *U*_21_ and *U*_22_, respectively. The mean payoff for PHs is represented by ${\bar{U}}_{2}$, while the replicator dynamic equation governing the behavior of PHs is given by *F*(*y*):

$$\begin{array}{c} U_{21}=x\left[\left(1-\phi \right)E+\left(1-\beta \right)R+Q_{2}\right]-C_{2}+\left(1-\alpha \right)S+W_{2}-Q_{2} \end{array}$$ (5)

$$\begin{array}{c} U_{22}=-xQ_{2}-\left(1-x\right)Q_{2}=-Q_{2} \end{array}$$ (6)

$$\begin{array}{c} {\bar{U}}_{2}=yU_{21}+\left(1-y\right)U_{22} \end{array}$$ (7)

$$\begin{array}{c} \begin{array}{cc} F(y) & =\frac{dy}{dt}=y\left(U_{21}-{\bar{U}}_{2}\right)\\ & =y\left(1-y\right)\left[x\left(1-\phi \right)E+x\left(1-\beta \right)R+xQ_{2}\right]-C_{2}+\left(1-\alpha \right)S+W_{2} \end{array} \end{array}$$ (8)

By setting *F*(*x*) = 0 and *F*(*y*) = 0, we can obtain $x^{*}=\frac{C_{2}-\left(1-\alpha \right)S-W_{2}}{\left(1-\phi \right)E+\left(1-\beta \right)R+Q_{2}}$ and $y^{*}=\frac{C_{1}-\alpha S-W_{1}}{\phi E+\beta R+Q_{1}}$, resulting in 5 equilibrium points from the following equation: O(0, 0), A(0, 1), B(1, 1), C(1, 0), and O*(x*, y*), where 0 < x* < 1 and 0 < y* < 1.

### Analysis of the equilibrium stability in the evolutionary game involving hierarchical hospitals within a medical alliance

According to research conducted by Selten [39] and Ritzberger [40], a strategy combination can only exhibit asymptotic stability in the replicator dynamic systems of evolutionary games if it constitutes a strict Nash equilibrium. This implies that for an equilibrium strategy combination to be asymptotically stable in an evolutionary game, it must necessarily be a strict Nash pure strategy. Furthermore, the method proposed by Friedman [41] can be employed to determine the evolutionary stability strategy of the set of dynamic equations through an analysis of the local stability exhibited by the Jacobian matrix system. First, the initial partial derivatives of the replicator dynamic equations for the hierarchical hospitals are computed individually to derive the Jacobian matrix *J*. Then, substituting the four points O, A, B and C into the Jacobian matrix yields the results of evolutionary stability for hierarchical hospitals. The Jacobian matrix *J* of the replicator dynamic Eqs (1) and (2) of the hierarchical hospitals is presented below:

$$\begin{array}{c} J=[\begin{array}{cc} \frac{\partial F(x)}{\partial x} & \frac{\partial F(x)}{\partial y}\\ \frac{\partial F(y)}{\partial x} & \frac{\partial F(y)}{\partial y} \end{array}] \end{array}$$ (9)

Where $\frac{\partial F(x)}{\partial x}=\left(1-2x\right)\left[y\left(\phi E+\beta R+Q_{1}\right)-C_{1}+\alpha S+W_{1}\right]$, $\frac{\partial F(x)}{\partial y}=x\left(1-x\right)\left(\phi E+\beta R+Q_{1}\right)$, $\frac{\partial F(y)}{\partial x}=y\left(1-y\right)\left[\left(1-\phi \right)E+\left(1-\beta \right)R+Q_{2}\right]$, $\frac{\partial F(y)}{\partial y}=\left(1-2y\right)\left[x\left(1-\phi \right)E+x\left(1-\beta \right)R+xQ_{2}-C_{2}+\left(1-\alpha \right)S+W_{2}\right]$.

The Jacobian matrix corresponding to the equilibrium point O(0, 0) is *J*_1_:

$$\begin{array}{c} J_{1}=[\begin{array}{cc} -C_{1}+\alpha S+W_{1} & 0\\ 0 & -C_{2}+\left(1-\alpha \right)S+W_{2} \end{array}] \end{array}$$ (10)

Where the corresponding eigenvalues are λ_1_ = −*C*_1_ + *αS* + *W*_1_ and λ_2_ = −*C*_2_ + (1 − *α*)*S* + *W*_2_.

The Jacobian matrix corresponding to the equilibrium point A(0, 1) is *J*_2_:

$$\begin{array}{c} J_{2}=[\begin{array}{cc} \phi E+Q_{1}-C_{1}+\alpha S+\beta R+W_{1} & 0\\ 0 & C_{2}-\left(1-\alpha \right)S-W_{2} \end{array}] \end{array}$$ (11)

Where the corresponding eigenvalues are λ_1_ = *ϕE* + *Q*_1_ − *C*_1_ + *αS* + *βR* + *W*_1_ and λ_2_ = *C*_2_ − (1 − *α*)*S* − *W*_2_.

The Jacobian matrix corresponding to the equilibrium point B(1, 1) is *J*_3_:

$$\begin{array}{c} J_{3}=[\begin{array}{cc} -\phi E-Q_{1}+C_{1}-\alpha S-\beta R-W_{1} & 0\\ 0 & -\left(1-\phi \right)E-Q_{2}+C_{2}-\left(1-\alpha \right)S-\left(1-\beta \right)R-W_{2} \end{array}] \end{array}$$ (12)

Where the corresponding eigenvalues are λ_1_ = −*ϕE* − *Q*_1_ + *C*_1_ − *αS* − *βR* − *W*_1_ and λ_2_ = −(1 − *ϕ*)*E* − *Q*_2_ + *C*_2_ − (1 − *α*)*S* − (1 − *β*)*R* − *W*_2_.

The Jacobian matrix corresponding to the equilibrium point C(1, 0) is *J*_4_:

$$\begin{array}{c} J_{4}=[\begin{array}{cc} C_{1}-\alpha S-W_{1} & 0\\ 0 & \left(1-\phi \right)E+Q_{2}-C_{2}+\left(1-\alpha \right)S+\left(1-\beta \right)R+W_{2} \end{array}] \end{array}$$ (13)

Where the corresponding eigenvalues are λ_1_ = *C*_1_ − *αS* − *W*_1_ and λ_2_ = (1 − *ϕ*)*E* + *Q*_2_ − *C*_2_ + (1 − *α*)*S* + (1 − *β*)*R* + *W*_2_.

According to the stability theorem of differential equations, the corresponding point in the game system is considered an evolutionary stable strategy (ESS) when all eigenvalues of the Jacobian matrix have negative real parts; conversely, if at least one eigenvalue is greater than zero, then the corresponding point represents an unstable equilibrium point in the game system. Through deductive reasoning, it can be inferred that equilibrium points O and B of the hierarchical hospital evolutionary system are ESS, while equilibrium points A and C represent unstable states. The local stability results of the evolutionary system for both THs and PHs are presented in Table 3.

**Table 3:** Local stability analysis of equilibrium points in the hierarchical hospital evolutionary system.

Dynamic equilibrium point	Eigenvalue λ_1_	Symbol	Eigenvalue λ_2_	Symbol	Stable point
O(0, 0)	−*C*_1_ + *αS* + *W*_1_	-	−*C*_2_ + (1 − *α*)*S* + *W*_2_	-	ESS
A(0, 1)	*ϕE* + *Q*_1_ − *C*_1_ + *αS* + *βR* + *W*_1_	+	*C*_2_ − (1 − *α*)*S* − *W*_2_	+	Unstable point
B(1, 1)	− *ϕE* − *Q*_1_ + *C*_1_ − *αS* − *βR* − *W*_1_	-	−(1 − *ϕ*)*E* − *Q*_2_ + *C*_2_ − (1 − *α*)*S* − (1 − *β*)*R* − *W*_2_	-	ESS
C(1, 0)	*C*_1_ − *αS* − *W*_1_	+	(1 − *ϕ*)*E* + *Q*_2_ − *C*_2_ + (1 − *α*)*S* + (1 − *β*)*R* + *W*_2_	+	Unstable point

Based on the findings presented in Table 3, the dynamic game system between the two parties is expected to converge at point O(0, 0) or point B(1, 1), depending on the anticipated benefits associated with the implementation of a remote consultation system for both entities. Although the cost of establishment exceeds the government subsidy, which can cause the system to converge to point O(0, 0), where both parties are reluctant to establish a remote consultation system, when the economic benefits derived from implementing such a system and its positive social impact outweigh the costs involved, convergence occurs at point B(1, 1), indicating mutual agreement in establishing a remote consultation system, thereby achieving smooth communication channels within the medical consortium and providing assistance to primary medical and health care.

### The initial strategy selection ratio influences the evolutionary game process between THs and PHs within a medical alliance

**Fig 3:**
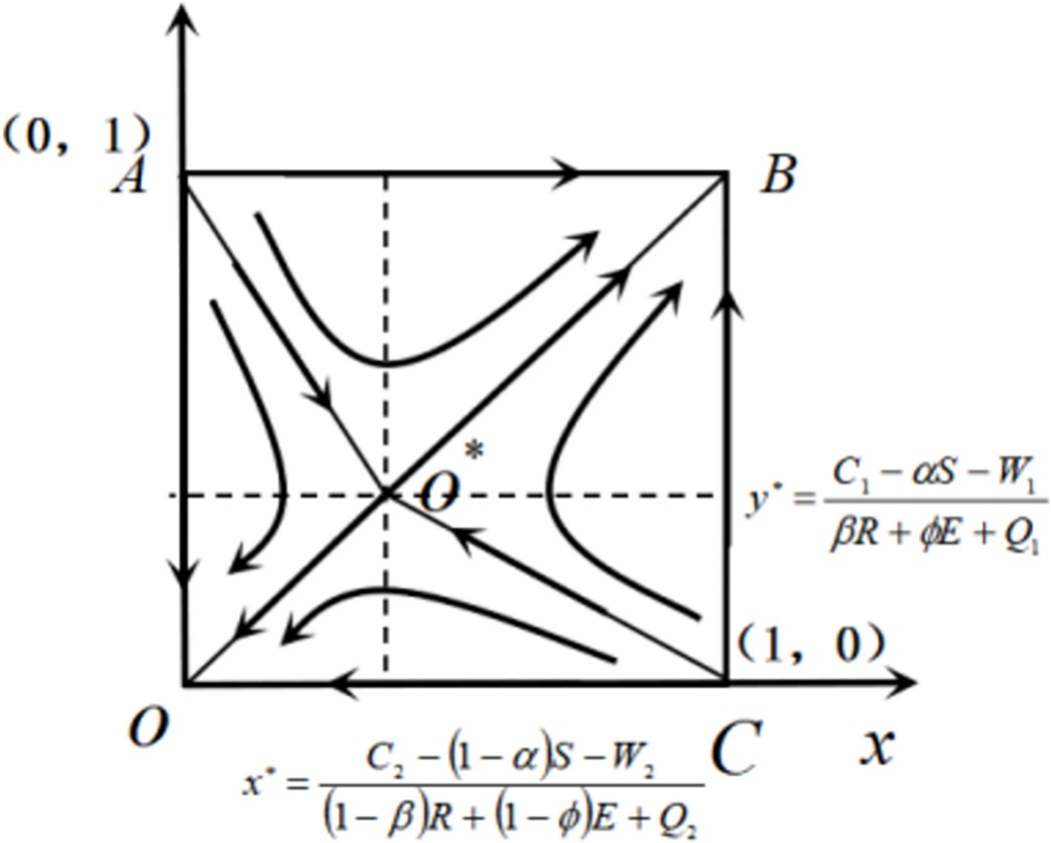
The trajectory of interactive dynamic evolution in establishing remote consultation systems between THs and PHs.

The local stability analysis of the equilibrium points in the evolutionary system between THs and PHs reveals that points O and B represent stable states of the system’s evolution, whereas points A and C are characterized as unstable. Fig 3 illustrates the trajectory of interactive dynamic evolution in the establishment of remote consultation systems between THs and PHs.

The evolution of the remote consultation system between THs and PHs can lead to two potential long-term outcomes: mutual adoption of the NERS strategy or mutual adoption of the ERS strategy. As shown in Fig 3, the initial evolution states of THs and PHs are located within quadrilateral AOCO*, where the initial probability *x* for THs to adopt the remote consultation system is less than x*, and the initial probability *y* for PHs to adopt the remote consultation system is less than y*. In this case, the long-term evolution of the system will ultimately converge to point O, indicating the eventual adoption of the NERS strategy by both sides. Instead, when the initial evolutionary state of THs and PHs lies within quadrilateral ABCO*, where the initial probability *x* for THs to choose to establish the remote consultation system exceeds x*, and the initial probability *y* for PHs to choose this system exceeds y*, the long-term evolution of the system will converge toward point B, indicating that both parties will eventually adopt an ERS strategy. Through this analysis, it can be inferred that the evolution of establishing a remote consultation system between THs and PHs within a medical alliance is influenced by the initial probabilities associated with strategy selection on both sides.

### The impacts of parameter changes on the evolutionary game process of THs and PHs in a medical alliance

The final selection of THs and PHs, as depicted in Fig 3, can be formulated as the problem of system convergence toward either point O or point B. To improve the effective downward allocation of high-quality medical resources within the medical alliances in China, both THs and PHs choose to establish a remote consultation system. The optimal outcome of the remote consultation channel formed by this is the downward allocation of medical resources, where both evolving systems ultimately converge at point B. This means that reducing quadrilateral AOCO* and increasing quadrilateral ABCO* are imperative in selecting optimal strategies for treating THs and PHs [42]. Therefore, the key problem in the selection of THs and PHs strategies can also be transformed into quadrilateral AOCO* and quadrilateral ABCO*. Meanwhile, as the area of quadrilateral AOCO* decreases, the area of quadrilateral ABCO* expands, ultimately leading to convergence at point B, where THs and PHs choose remote consultation. Therefore, we calculate the area of the quadrilateral AOCO* to determine the parameters that affect the outcome of the evolutionary game. The formula to calculate the area of quadrilateral AOCO* can be expressed as follows:

$$\begin{array}{c} \begin{array}{cc} S_{AOCO^{*}} & =S_{AOO^{*}}+S_{OO^{*}C}\\ & =\frac{1}{2}\times \left[\frac{C_{2}-\left(1-\alpha \right)S-W_{2}}{\left(1-\beta \right)R+\left(1-\phi \right)E+Q_{2}}+\frac{C_{1}-\alpha S-W_{1}}{\phi E+\beta R+Q_{1}}\right] \end{array} \end{array}$$ (14)

Using MATLAB 2018a, we calculate the first partial derivative of a parameter that influences the quadrilateral AOCO* area. Subsequently, we analyze how variations in these parameters impact the evolutionary game dynamics between THs and PHs within a medical alliance.

#### Establishing costs *C*_1_/*C*_2_

$$\begin{array}{c} \frac{\partial S_{AOCO^{*}}}{\partial C_{1}}=\frac{1}{2}\times \frac{1}{Q_{1}+\phi E+\beta R}>0 \end{array}$$ (15)

$$\begin{array}{c} \frac{\partial S_{AOCO^{*}}}{\partial C_{2}}=\frac{1}{2}\times \frac{1}{Q_{2}+\left(1-\phi \right)E+\left(1-\beta \right)R}>0 \end{array}$$ (16)

The first partial derivatives of *S*_AOCO*_ with respect to *C*_1_ and *C*_2_ are both positive, indicating that *S*_AOCO*_ exhibits a monotonically increasing behavior in response to changes in *C*_1_ and *C*_2_. As the values of *C*_1_ and *C*_2_ increase, the probability of the system converging toward point O (0, 0) also increases, while the probability of both sides choosing establishment decreases. Therefore, when the cost of implementing a remote consultation system decreases, THs and PHs are more inclined to adopt the ERS strategy, thus increasing the likelihood of the effective downward allocation of high-quality medical resources within medical alliances in China.

#### Government subsidies *S*

$$\begin{array}{c} \begin{array}{cc} \frac{\partial S_{AOCO^{*}}}{\partial S} & =\frac{1}{2}\times \left[\frac{\alpha -1}{Q_{2}+\left(1-\phi \right)E+\left(1-\beta \right)R}-\frac{\alpha }{Q_{1}+\phi E+\beta R}\right]<0 \end{array} \end{array}$$ (17)

The first partial derivative of *S* with respect to *S*_AOCO*_ is less than 0, signifying that *S*_AOCO*_ is a monotonic decreasing function of *S*. *S*_AOCO*_ increases as *S* increases, the probability of the system evolving to point O (0, 0) increases, and the probability of both sides choosing ERS decreases, so the probability of the system evolving to point B (1, 1) increases. This indicates that when government subsidies are sufficient, the system evolution result is (ERS, ERS).

#### Economic benefits *R*

$$\begin{array}{c} \begin{array}{cc} \frac{\partial S_{AOCO^{*}}}{\partial R} & =\frac{\left(\beta -1\right)[C_{2}-W_{2}+\left(\alpha -1\right)S]}{2{\left[\left(\phi -1\right)E+\left(\beta -1\right)R-Q_{2}\right]}^{2}}+\frac{\beta (W_{1}-C_{1}+\alpha S)}{2{\left(\beta R+\phi E+Q_{1}\right)}^{2}}<0 \end{array} \end{array}$$ (18)

The first partial derivative of *R* with respect to *S*_AOCO*_ is less than 0, which shows that *S*_AOCO*_ is a monotonic decreasing function of *R*. *S*_AOCO*_ increases as *R* increases, the probability of the system evolving to point O (0, 0) increases, and the probability of both sides choosing ERS decreases, so the probability of the system evolving to point B (1, 1) increases. This indicates that when the benefits produced by both parties after establishing remote consultation are more common, the system evolution result is more likely to be (ERS, ERS).

#### Social benefits *W*_1_/*W*_2_

$$\begin{array}{c} \frac{\partial S_{AOCO^{*}}}{\partial W_{1}}=-\frac{1}{2}\times \frac{1}{Q_{1}+\phi E+\beta R}<0 \end{array}$$ (19)

$$\begin{array}{c} \frac{\partial S_{AOCO^{*}}}{\partial W_{2}}=-\frac{1}{2}\times \frac{1}{Q_{2}+\left(1-\phi \right)E+\left(1-\beta \right)R}<0 \end{array}$$ (20)

The negative first partial derivatives of *S*_AOCO*_ with respect to *W*_1_ and *W*_2_ demonstrate that *S*_AOCO*_ is a monotonic decreasing function with respect to both *W*_1_ and *W*_2_. As the values of *W*_1_ and *W*_2_ increase, *S*_AOCO*_ decreases, leading to an increase in the probability of the system evolving toward point B (1, 1), where both sides choose ERS. Therefore, when the social benefits derived from implementing a remote consultation system are maximized, the evolutionary outcome of the system can be optimized, that is, the two parties choose (ERS, ERS).

#### The resource sinking utility *E*

$$\begin{array}{c} \begin{array}{cc} \frac{\partial S_{AOCO^{*}}}{\partial E} & =\frac{\left(\phi -1\right)[C_{2}-\left(1-\alpha \right)S-W_{2}]}{2{\left[\left(\phi -1\right)E+\left(\beta -1\right)R-Q_{2}\right]}^{2}}+\frac{\phi (W_{1}-C_{1}+\alpha S)}{2{\left(\beta R+\phi E+Q_{1}\right)}^{2}}<0 \end{array} \end{array}$$ (21)

The negative first-order partial derivative of *E* with respect to *S*_AOCO*_ implies that *E* exhibits a monotonic decreasing behavior in response to increasing values of *S*_AOCO*_, suggesting a decrease in *E* as *S*_AOCO*_ increases. Consequently, this leads to an increased probability of the system evolving toward point B (1, 1), where both sides opt for establishment. Therefore, in the absence of any obstacles to the remote consultation channel and with both parties able to access a greater resource sinking utility, the evolutionary outcome of the system is determined as (ERS, ERS).

#### Reputational damage and adverse consequences *Q*_1_/*Q*_2_

$$\begin{array}{c} \frac{\partial S_{AOCO^{*}}}{\partial Q_{1}}=\frac{W_{1}-C_{1}+\alpha S}{2{\left(Q_{1}+\phi E+\beta R\right)}^{2}}<0 \end{array}$$ (22)

$$\begin{array}{c} \frac{\partial S_{AOCO^{*}}}{\partial Q_{2}}=\frac{-C_{2}+\left(1-\beta \right)R+W_{2}}{2{\left[\left(\phi -1\right)E+\left(\beta -1\right)R-Q_{2}\right]}^{2}}<0 \end{array}$$ (23)

The first-order partial derivatives of *S*_AOCO*_ with respect to *Q*_1_ and *Q*_2_ are both negative, revealing that *S*_AOCO*_ is a monotonic decreasing function of *Q*_1_ and *Q*_2_. As the values of *Q*_1_ and *Q*_2_ increase, the probability of the system evolving toward point B (1, 1) also increases. Therefore, in cases where the remote consultation channel encounters obstacles, the amplification of reputational damage and adverse consequences resulting from complaints and inadequate performance by THs and PHs renders the adoption of the ERS strategy more probable.

#### Distribution proportion of government subsidies *α*, distribution proportion of economic benefit *β*, and the proportion of distribution of utility derived from the downward allocation of resources *ϕ*

$$\begin{array}{c} \begin{array}{cc} \frac{\partial S_{AOCO^{*}}}{\partial \alpha } & =\frac{S}{2[\left(\beta -1\right)R+\left(\phi -1\right)E-Q_{2}]}-\frac{S}{2(\beta R+\phi E+Q_{1})}\\ & =\frac{S(E-Q_{1}-Q_{2}+R-2\phi E-2\beta R)}{2\left[\left(\beta -1\right)R+\left(\phi -1\right)E-Q_{2}\right]\left(\beta R+\phi E+Q_{1}\right)} \end{array} \end{array}$$ (24)

$$\begin{array}{c} \begin{array}{cc} \frac{\partial S_{AOCO^{*}}}{\partial \beta } & =\frac{R(W_{1}-C_{1}+\alpha S)}{2{\left(Q_{1}+\phi E+\beta R\right)}^{2}}+\frac{R[C_{2}-W_{2}+\left(\alpha -1\right)S]}{2{\left[\left(\beta -1\right)R+\left(\phi -1\right)E-Q_{2}\right]}^{2}}\\ & =\frac{R}{2}\times \left[\frac{-y^{*}}{Q_{1}+\phi E+\beta R}-\frac{x^{*}}{\left(\beta -1\right)R+\left(\phi -1\right)E-Q_{2}}\right] \end{array} \end{array}$$ (25)

$$\begin{array}{c} \begin{array}{cc} \frac{\partial S_{AOCO^{*}}}{\partial \phi } & =\frac{E(W_{1}-C_{1}+\alpha S)}{2{\left(Q_{1}+\phi E+\beta R\right)}^{2}}+\frac{E[C_{2}-W_{2}+\left(\alpha -1\right)S]}{2{\left[\left(\beta -1\right)R+\left(\phi -1\right)E-Q_{2}\right]}^{2}}\\ & =\frac{E}{2}\times \left[\frac{-y^{*}}{Q_{1}+\phi E+\beta R}-\frac{x^{*}}{\left(\beta -1\right)R+\left(\phi -1\right)E-Q_{2}}\right] \end{array} \end{array}$$ (26)

From Eq (24), we can see that [(*β* − 1)*R* + (*ϕ* − 1)*E* − *Q*_2_] < 0 is always true, so when *S*(*E* − *Q*_1_ + *Q*_2_ + *R* − 2*ϕE* − 2*βR*) > 0, $\frac{\partial S_{AOCO^{*}}}{\partial \alpha }>0$ is derived. This part of the formula can be analyzed through the optimal value method; that is, if *Min*[*S*(*E* − *Q*_1_ + *Q*_2_ + *R* − 2*ϕE* − 2*βR*)] > 0 is satisfied, then −*Q*_1_ + *Q*_2_ − *R* − *E* > 0 is derived because the values of *ϕ* and *β* are both located at (0, 1), thus, *Q*_2_ − *Q*_1_ > *E* + *R* is obtained. Since *E* and *R* are both greater than zero, it is finally concluded that when *Q*_1_ < *Q*_2_, $\frac{\partial S_{AOCO^{*}}}{\partial \alpha }>0$ is established. This indicates that *S*_AOCO*_ exhibits monotonically increasing behavior in response to changes in *α*. As *α* increases, the probability of the system converging toward point O (0, 0) also increases, while the probability of both sides opting for establishment decreases. Conversely, when *S*(*E* − *Q*_1_ + *Q*_2_ + *R* − 2*ϕE* − 2*βR*) < 0, $\frac{\partial S_{AOCO^{*}}}{\partial \alpha }<0$ is true. In the same way, when *Max*[*S*(*E* − *Q*_1_ + *Q*_2_ + *R* − 2*ϕE* − 2*βR*)] < 0 is satisfied, the values of *β* and *ϕ* are both located at (0,1), and then, −*Q*_1_ + *Q*_2_ − *R* − *E* < 0 is derived; thus, *Q*_2_ − *Q*_1_ < *E* + *R* is obtained. Since *E* and *R* are both greater than zero, $\frac{\partial S_{AOCO^{*}}}{\partial \alpha }<0$ is finally proven when *Q*_1_ > *Q*_2_, indicating that *S*_AOCO*_ is a monotonic decreasing function of *α*, *S*_AOCO*_ decreases with increasing *α*, and the probability of system evolution to B (1, 1) increases; that is, both sides are more inclined to choose ERS. Therefore, when the reputation loss and negative impact of THs not establishing remote consultation are smaller than those of PHs, the strategic choices of both parties will evolve according to the increase in government subsidies obtained by THs. In contrast, when the reputation loss and negative impact of THs not establishing remote consultation are greater than those of PHs, both parties will eventually choose to establish a remote consultation system with the increase in government subsidies received by THs.

By substituting x* and y* into Eqs (25) and (26), the same extreme value method is used for analysis, and the threshold value *Q*_1_ − *Q*_2_ < *E* + *R* can be obtained; that is, when *Q*_1_ < *Q*_2_ is satisfied, $\frac{\partial S_{AOCO^{*}}}{\partial \beta }>0$ and $\frac{\partial S_{AOCO^{*}}}{\partial \phi }>0$ are valid, suggesting that *S*_AOCO*_ is a monotonic increasing function of *β* and *ϕ*, which increases with increasing *β* and *ϕ*, and the probability of the system evolving to point B(1, 1) decreases; that is, the probability of both sides choosing NERS increases. In contrast, when −*Q*_2_ < *E* + *R*, that is, |−*Q*_1_| > |*E* + *R*|, $\frac{\partial S_{AOCO^{*}}}{\partial \phi }<0$ hold, *S*_AOCO*_ is a monotonic decreasing function of *β* and *ϕ*, which decreases with increasing *β* and *ϕ*, and the probability of system evolution to point B (1, 1) increases; that is, both sides are more inclined to choose ERS. Therefore, when THs do not establish a remote consultation system and the reputation loss in the patient group is smaller than that of PHs, the strategic choice of both parties will evolve to not establish a remote consultation system according to the economic benefits gained by THs and the increase in resource sinking utility. At the same time, the reputation loss of PHs in the patient group due to the absence of a remote consultation system is greater than the sum of the economic benefits and resource sinking utility obtained by both parties after the construction of a remote consultation system, and both parties therefore tend to construct a remote consultation system.

Accordingly, with the decrease in reputation suffered by THs and PHs, changes in the government subsidy distribution ratio, economic income distribution ratio, and medical resource sinking acquisition ratio will affect the final choices of both sides. This indicates that THs and PHs are very concerned about the reputation loss and negative impact caused by the failure to establish a remote consultation system in the patient group and that the proportions of government subsidy distribution, economic income distribution, and medical resource sinking can compensate for the reputation loss and negative impact to a certain extent.

## Numerical simulation

To further validate the practicality and applicability of the evolutionary game model, this study conducted numerical simulations using MATLAB 2018a. Key parameters were selected to visually depict the evolution process and the equilibrium state of the choices made by THs and PHs to establish a remote consultation system. These parameters encompass the initial proportions of the THs and PHs choosing the ERS strategy and the influence of parameter modifications on the interactive behavior of both parties. By referring to existing simulation values [27, 30, 31, 42, 43] and in accordance with the research hypotheses, specific values for each parameter were assigned as presented in Table 4 while simultaneously satisfying conditions 0 < x* < 1 and 0 < y* < 1.

**Table 4:** Table of parameter assignments.

*C*_1_	*C*_2_	*S*	*R*	*W*_1_	*W*_2_	*E*	*Q*_1_	*Q*_2_	*α*	*β*	*ϕ*
7	6	3	4	2	1	5	1	2	0.5	0.5	0.5

**Fig 4:**
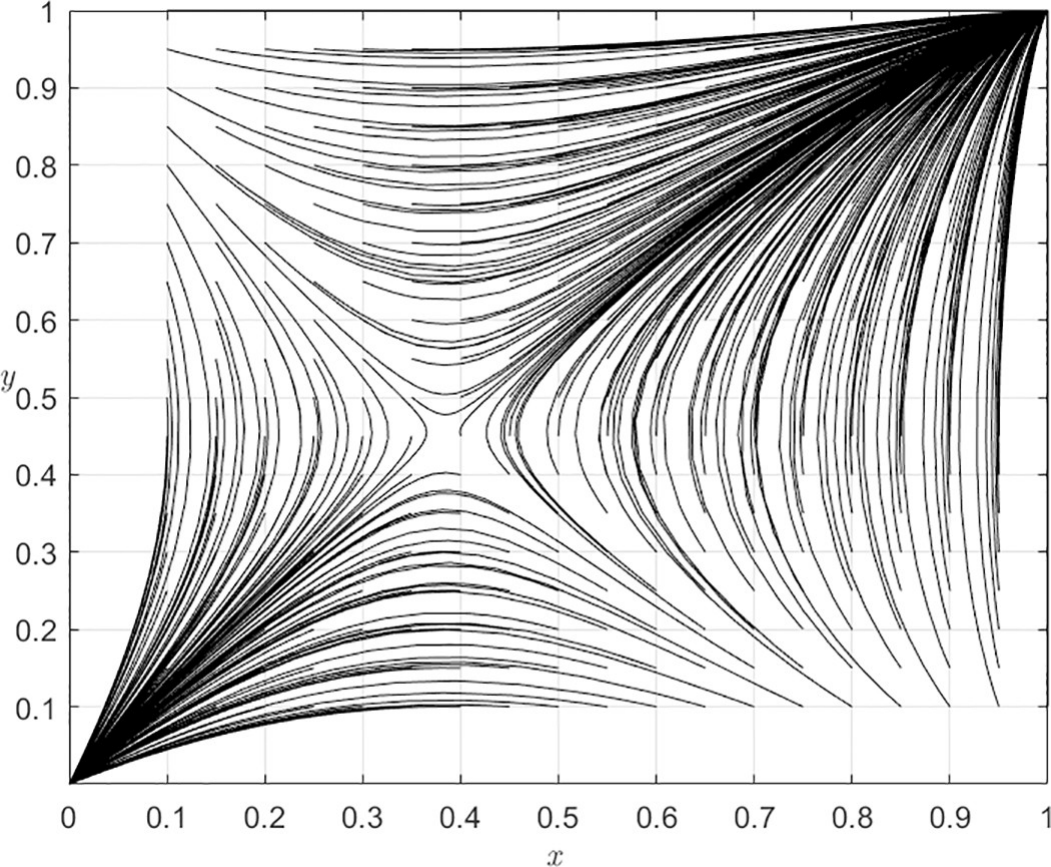
The dynamic evolutionary trends of establishing remote consultation systems in THs and PHs.

The computed values of (x*, y*) are determined to be (0.38, 0.45), while the numerical simulation results are depicted in Fig 4. The system is approximately bounded by the intervals {0.3< *x* <0.4} ∩ {0.4< *y* <0.5} and asymptotically approaches the points (0, 0) and (1, 1). The results demonstrate the validity of the earlier mathematical deduction regarding the stability of system evolution, indicating that the final strategy choices made by both players are not solely determined by their own initial strategy proportions but also influenced by the initial strategy proportions of other players in the game.

Furthermore, alterations in the parameter values also influence the temporal resolution and outcomes of system evolution. Given that (x*, y*) was previously determined to be (0.38, 0.45) in the preceding analysis, for convenience in the discussion, we assume equal initial probabilities of THs selecting ERS (*x*) and PHs choosing ERS (*y*) at 0.5 to examine the impact of each parameter on the process and outcomes of system evolution.

### Changes in the resource sinking utility *E*

**Fig 5:**
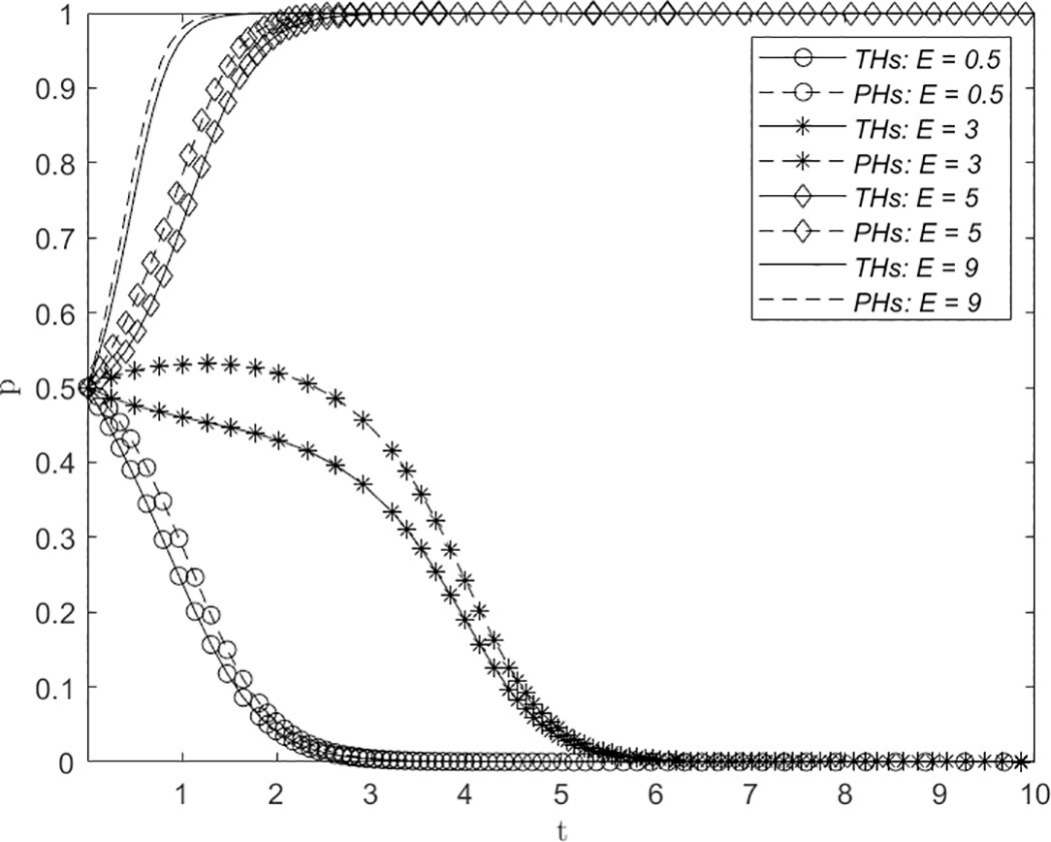
The impact of changes in the resource sinking utility *E* on system evolution.

The impact of changes in the resource sinking utility *E* on system evolution is illustrated in Fig 5. *E* values of 0.5, 3, 5, and 9 correspond to the levels of resource sinking utility for THs and PHs classified as very low, low, medium, and high, respectively. The results demonstrate that for *E* = 0.5, the system converges to point (0, 0), with both THs and PHs adopting the NERS strategy and stabilizing after 4 evolution steps. When *E* = 3, the system also converges to point (0, 0), with both THs and PHs favoring the NERS strategy and reaching stability after 6 evolution steps. However, PHs initially tend to lean toward selecting the ERS strategy. When *E* is 5 or 9, the system converges to point (1, 1), with both THs and PHs adopting the ERS strategy and stabilizing rapidly at step lengths ranging between 2∼3 and 1∼2, respectively. This highlights the significance of resource sinking utility in the establishment of a remote consultation system between THs and PHs. When the resource sinking utility generated by an unobstructed remote consultation channel is greater, the convergence toward consensus of the ERS strategy between both parties is faster.

### Changes in reputational damage and adverse consequences *Q*_1_/*Q*_2_

**Fig 6:**
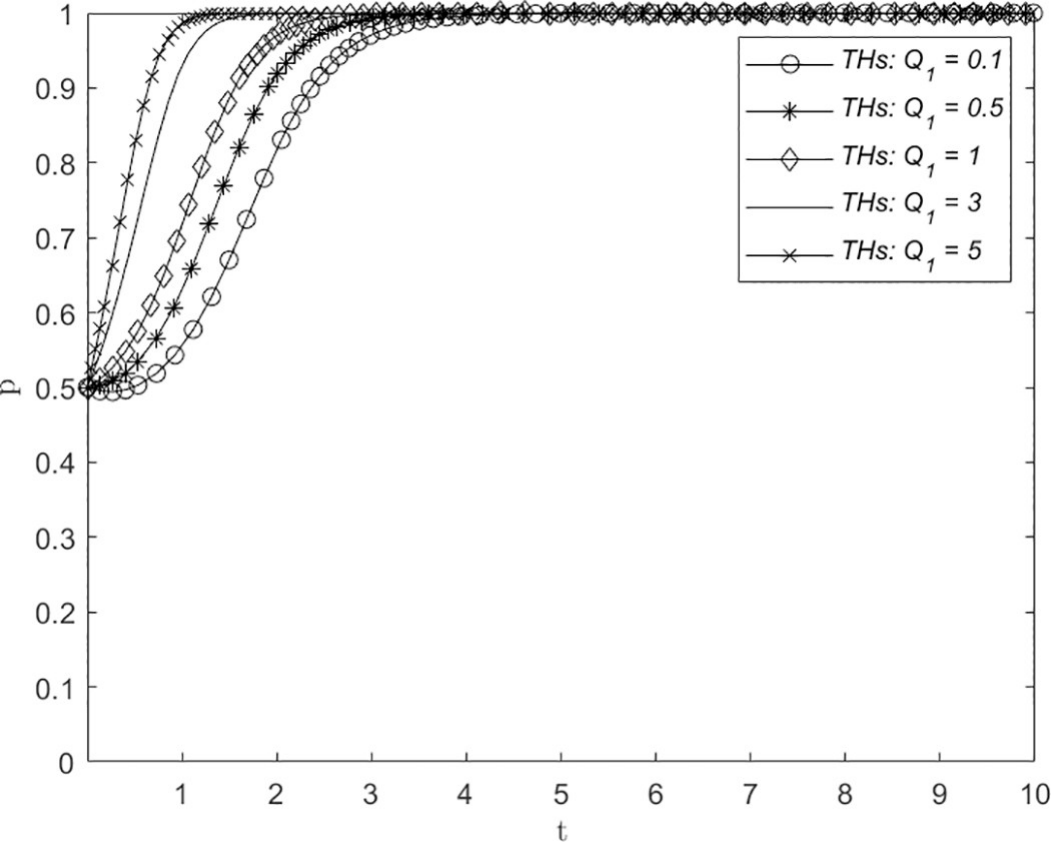
The impact of changes in reputational damage and adverse consequences *Q*_1_ on system evolution.

**Fig 7:**
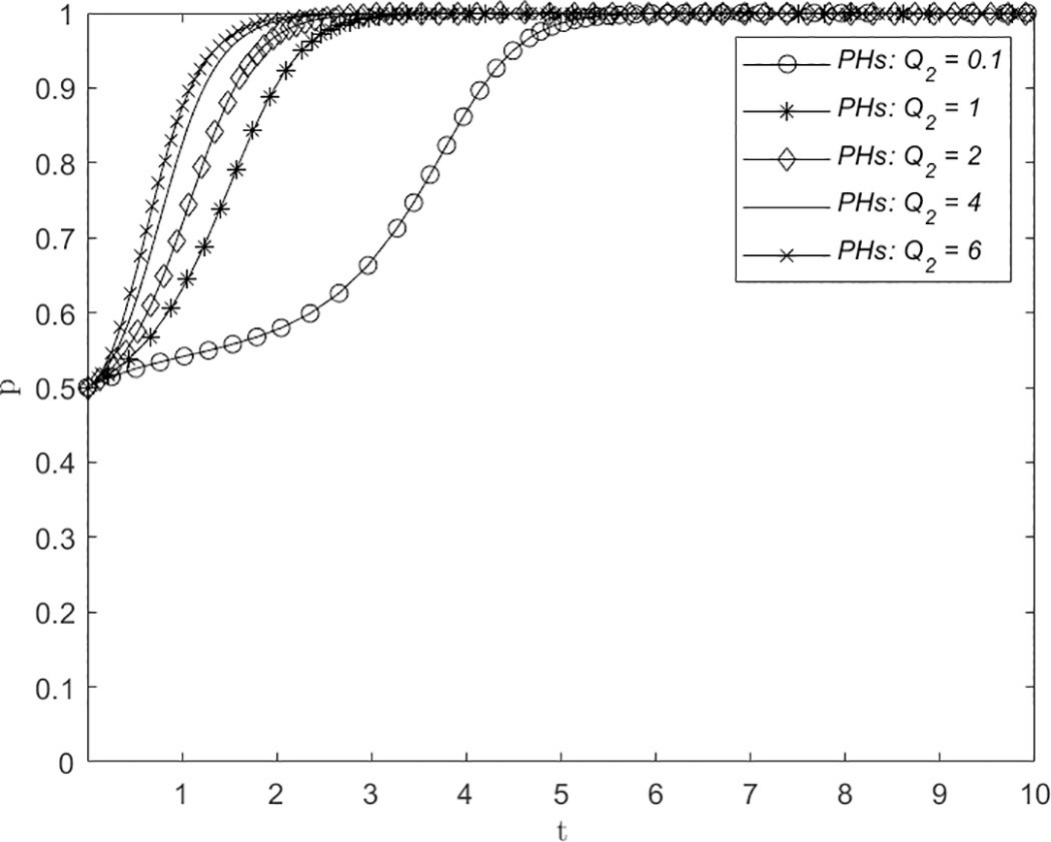
The impact of changes in reputational damage and adverse consequences *Q*_2_ on system evolution.

Figs 6 and 7 illustrate the influence of variations in reputational damage and adverse consequences *Q*_1_/*Q*_2_ on the strategic decision-making process of THs and PHs, respectively. The assigned values of *Q*_1_/*Q*_2_ are 0.1, 0.5, 1, 3, and 5, representing low, medium, and high levels of reputational damage and adverse consequences suffered by THs and PHs due to the absence of a remote consultation system. The numerical simulation results of both figures indicate that the system will converge to point (1, 1) irrespective of the level of reputational damage and adverse consequences. Both THs and PHs choose the ERS strategy, although with varying probability fluctuations and convergence times. The step length for THs to adopt the ERS strategy decreases as *Q*_1_ increases, as shown in Fig 6. However, when *Q*_1_ is 3 or 5, the number of evolutionary steps remain roughly constant at approximately 2. Similarly, in Fig 7, as *Q*_2_ increases, the step length for PHs to adopt the ERS strategy decreases. However, during the initial stages of evolution, PHs exhibit a propensity for selecting the NERS strategy. This finding suggests that both THs and PHs place significant emphasis on reputational damage and adverse consequences, as they tend to prioritize establishment even when the extent of reputation loss is minimal. However, notably, the evolutionary steps of PHs selecting ERS were significantly larger than those of THs in the early stages of evolution for low-intensity reputation loss but medium- and high-intensity reputation loss did not significantly improve the evolutionary rate of THs and PHs. Therefore, it is necessary to reasonably control the influence of this parameter on the establishment of a remote consultation system and strive to achieve the most significant effect.

### Changes in the proportion of distribution of government subsidies *α*, the proportion of distribution of economic benefits *β*, and the proportion of distribution of utility derived from the downward allocation of medical resources *ϕ*

**Fig 8:**
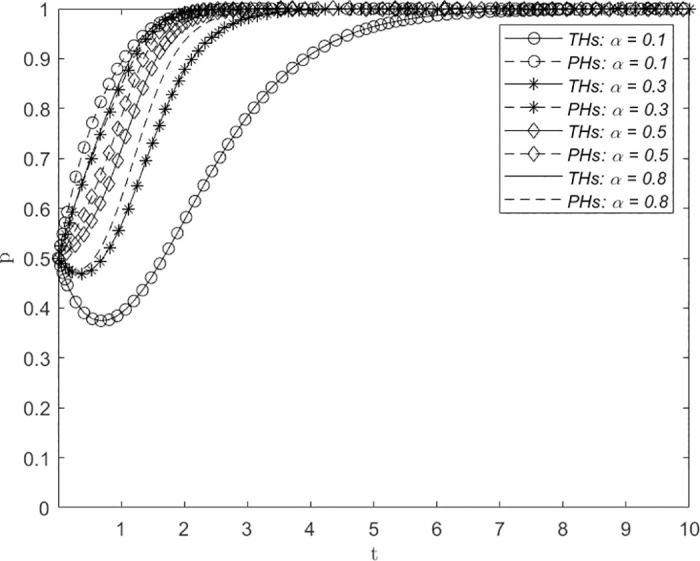
The impact of changes in the distribution proportion of government subsidies *α* on system evolution.

**Fig 9:**
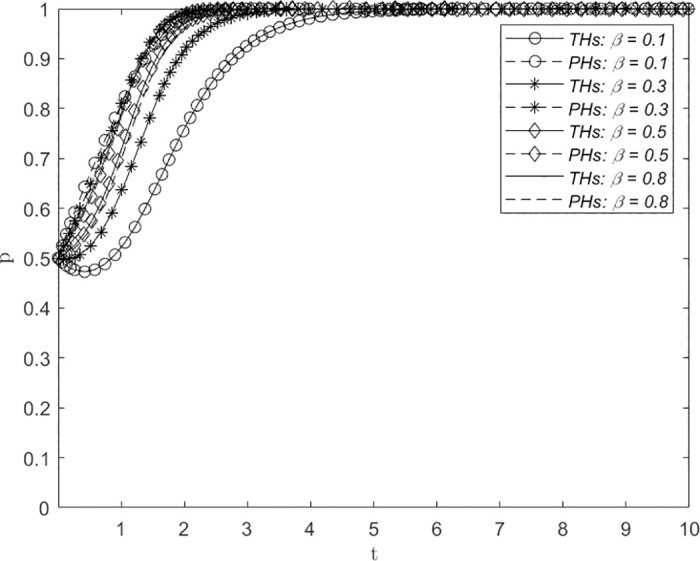
The impact of changes in the economic benefit allocation ratio *β* on system evolution.

**Fig 10:**
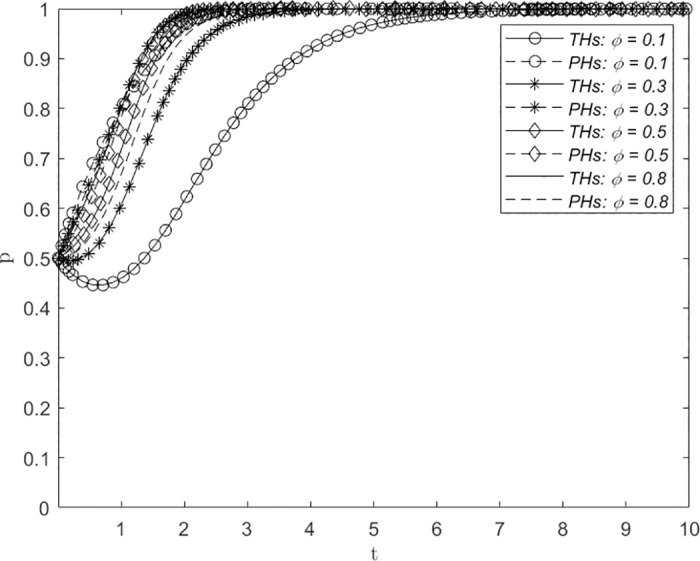
The impact of changes in the proportion of effectiveness in utility derived from the downward allocation of medical resources *ϕ* on system evolution.

Figs 8–10 depict the influence of variations in the proportion of distribution of government subsidies *α*, the proportion of distribution of economic benefits *β*, and the proportion of distribution of utility derived from the downward allocation of medical resources *ϕ* on the evolution of the system. The assigned values are 0.1, 0.3, 0.5 and 0.8, representing extreme inequality (strongly biased toward PHs), inequality, balance, and extreme inequality (strongly biased toward THs), respectively, in the proportion of the distribution of government subsidies for THs and PHs. The figures demonstrate that with increasing values of *α*, *β* and *ϕ*, the number of evolutionary steps required for THs and PHs to consistently select the ERS strategy showed a trend of first decreasing and then increasing. Initially, this convergence decreases, followed by an increase with a turning point at approximately 0.5. This suggests that a greater balance of *α*, *β* and *ϕ* leads to a shorter time required for THs and PHs to reach a decision regarding the establishment of a remote consultation system, thereby facilitating faster adoption of the ERS strategy by both parties.

### The variables *α*, *β* and *ϕ* undergo simultaneous changes

**Fig 11:**
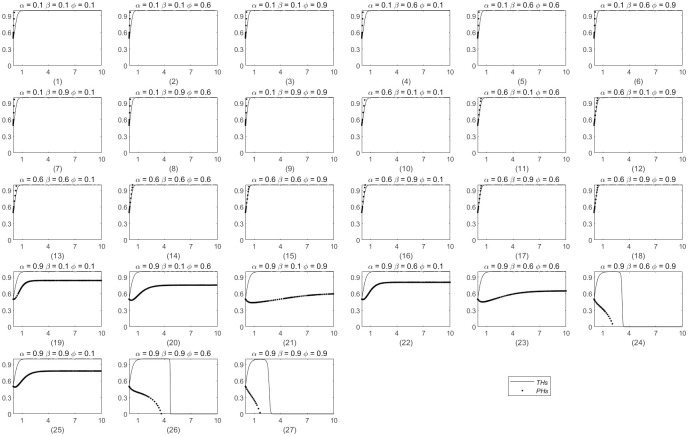
The combined influence of simultaneous modifications *α*, *β* and *ϕ* on the system’s evolutionary trajectory.

The above analysis demonstrates that individual changes in *α*, *β* and *ϕ* exert a significant influence on the process and outcomes of system evolution. To better align the practical implementation of a remote consultation system between THs and PHs and to address the limitations arising from individual modifications of *α*, *β* and *ϕ*, measurements are simultaneously captured at 0.1, 0.6, and 0.9 to represent the highly imbalanced (strongly biased toward PHs), relatively equitable, and highly imbalanced (strongly biased toward THs) distribution ratios of government subsidies, economic benefits, and utility derived from the downward allocation of medical resources, respectively. The three ratios in the three states are arranged and combined, resulting in the 27 evolutionary outcomes depicted in Fig 11, which are used to analyze the evolution process and outcomes of the system under simultaneous changes in *α*, *β* and *ϕ*. In Fig 11, the horizontal axis represents the evolution step size, the vertical axis represents the probability of both sides choosing ERS, the straight black line represents the THs, and the black dots and lines represent the PHs. As illustrated in Fig 11, the 27 evolutionary outcomes can be organized into three categories.

In the first type (1) ∼ (18), both sides choose the ERS strategy, and the evolutionary system reaches equilibrium. According to the evolution trend and the time step required by the evolution result, the situation can be divided into the following two situations. 1) In results (1) ∼ (9), when the value of *α* is 0.1, the proportion of government subsidies is completely biased toward PHs, and PHs are always close to the vertical axis and directly choose the ERS strategy without hesitation. THs also choose the ERS strategy, but the stabilization time is longer than that of PHs, indicating that despite these three kinds of returns and very low conditions, THs are still willing to establish a remote consultation system because they believe that the social benefits after completion are great, which is consistent with the public welfare of public hospitals in China. At the same time, with the continuous increase in *β* and *ϕ*, that is, when the proportion of economic income distribution and the proportion of resource subsidence utility acquisition slowly increase to THs, the evolution step of THs decreases from 1 to 0 ∼ 1, showing a downward trend, which also means that the time required for both sides to reach an agreement decreases. This indicates that government subsidies are very attractive to PHs in the initial stage of establishing remote consultation. 2) According to results (10) ∼ (18), as *α* increases to 0.6, THs receive more government subsidies than PHs, but the distribution ratio between the two sides is still relatively balanced. The evolution step of PHs slowly moves from the vertical axis of infinite sticking, this is 0 to 1, and the evolution trend of THs and PHs approaches infinity and finally overlaps. This shows that the time needed for the two sides to reach an agreement is shorter and that they reach an agreement directly in the early stage of evolution, which suggests that both sides are satisfied with the distribution result.

The second category includes (19) ∼ (23) and (25), which cannot reach evolutionary equilibrium. When the value of *α* is 0.9, the proportion of government subsidies is completely biased toward THs, and THs always choose the ERS strategy. However, despite the continuous increase in *β* and *ϕ*, PHs preferred the NERS strategy in the early stage of evolution and prefer the ERS strategy in the middle and later stages. However, in terms of the probability of choice, the final probability of THs is 1, and PHs finally stabilize at 0.6, which does not reach the optimal equilibrium of the evolutionary system (1, 1). This is a negative confirmation of result 2) in the first category; when the three income distributions are not balanced, the two parties cannot reach the optimal equilibrium. Notably, the probability of PHs choosing the ERS strategy in (19), (20), (22), and (25) is higher than the initial probability, and the final probability of (19) is the largest. Compared with (20) and (22), when the government subsidy is constant, an increase in the resource sinking utility and economic benefit can improve the probability of PHs choosing the ERS strategy, and the positive effect of the resource sinking utility is more obvious than that of the economic benefit in the middle and later stages of evolution.

The third category includes (24), (26), and (27). When *α* is 0.9, the government subsidy distribution is completely biased toward THs, while when *β* and *ϕ* are 0.6 or 0.9, the economic income distribution and resource sinking utility are biased toward THs. Finally, although the evolutionary system reaches equilibrium, both sides choose the NERS strategy. Different from the THs picking the ERS strategy when the total revenue of THs is completely biased to PHs in (1), THs choose the NERS strategy when PHs receive very little revenue. This fully shows that PHs are in a weak position in the establishment of remote consultation in medical alliances and need more income as a guarantee for choosing the ERS strategy.

Accordingly, through the analysis of different permutations and combinations of *α*, *β* and *ϕ*, the following were found: (1) The social benefits of THs selecting ERS are enough to compensate for their lack of other benefits, and they are the defenders of the public welfare characteristics of public hospitals in China. (2) When the income distribution is more balanced, the time for THs and PHs to approach the ERS strategy is shorter. (3) Compared with THs, PHs are weak. The government needs to ensure that PHs have enough income to promote system evolution to the optimal equilibrium point (1, 1) and that PHs can receive enough government subsidies in the initial stage of establishing remote consultation. In the middle and later stages, more attention should be given to the decreasing utility of medical resources obtained by PHs and ensuring their economic income.

## Discussion

On the basis of considering the resource sinking effect brought by the remote consultation channel, this paper constructs an evolutionary game model of THs and PHs in a medical alliance for establishing a remote consultation system, analyzes the equilibrium state and evolution process of the game by replicating the dynamic equation, and further verifies the correctness of the mathematical derivation by using a numerical simulation. The results show that the initial proportion of the ERS strategy selected by THs and PHs, the cost of establishment, the proportion of the government subsidy distribution, the proportion of the economic income distribution, the proportion of the resource sinking utility, and reputation loss are the main factors. This is consistent with the research results of references [27–29, 42]; that is, only under the basic condition that the total benefit is greater than the total cost do game players tend to choose to establish or use a remote consultation system. In addition, according to the literature [43], the emphasis on patient subsidies can better encourage the two sides to reach the optimal solution, which is similar to the research results obtained in this paper that the ratio of resource subsidence utility acquisition is more inclined to PHs, and both are disadvantaged parties that give more attention to the evolutionary system. At the same time, the research results of this paper are different. For example, the research results of this paper suggest that for the reputation loss caused by THs and PHs not establishing a remote consultation system, the punishment from the government should be moderate, which is different from the research conclusion in the literature of increasing the punishment for doctors who provide inferior information [30]. In addition, one article [31] argues that the final choice of game players will be significantly affected by free riding, which is not taken into account in this paper and is the topic of future research. Therefore, the research results of this paper are not inconsistent with most of the research results, and at the same time, they have their own unique views, such as that THs and PHs should suffer a certain range of reputation loss; otherwise, the effect of punishment will not be achieved. Compared with the allocation ratio of financial subsidies and economic income, the access ratio of the medical resource sinking utility has a more significant impact on the strategy selection of THs and PHs. This is also consistent with the Chinese government’s efforts to continuously promote the reform of the medical and health system to improve the pattern of medical treatment and promote the sinking of high-quality medical resources.

## Conclusion and management insights

### Conclusion

These research results have important practical significance for promoting the construction of remote consultations in medical alliances in China. The main conclusions are as follows: (1) Government subsidies, economic benefits, and the utility of resource sinks are important factors for THs and PHs in establishing a remote consultation system. (2) Reputation loss has a certain regulatory effect on the behavior of THs and PHs. (3) The balance of the government subsidy distribution ratio, economic income distribution ratio, and resource sinking utility acquisition ratio is directly related to the steps required for THs and PHs to reach consistency between the final strategy selection and the final strategy trend. When the ratio is more balanced, the time required for the system to evolve to ERS is shorter. (4) In the early stage of the construction of the remote consultation system, policy subsidies have a certain impact on the decision-making of both sides. However, in the long run, PHs give more attention to the resource sinking utility caused by remote consultation channels and economic benefits.

### Management insights

According to the previous research conclusions, the following management implications are drawn.

(1) In terms of government subsidy support, it is essential to appropriately prioritize PHs to ensure the financial stability of both types of hospitals. In terms of economic benefits, it is imperative for the relevant departments to refine pricing mechanisms to ensure that hospitals generate fundamental income after the establishment of remote consultation systems. Furthermore, enhancing the benefit distribution mechanism is crucial for ensuring an equitable allocation of income among hospitals [44]. In terms of resource sinking efficiency, it is crucial to ensure the exclusive utilization of remote consultation channels and establish a clear delineation of responsibilities to guide the transfer of high-quality medical resources from THs to PHs once the channel becomes unobstructed. The functions and roles of the remote consultation system within the medical alliance in China can be formally consulted in writing to ensure its effectiveness as a carrier to achieve the downward allocation of high-quality medical resources, reduce the decision-making time for both parties, and remove valuable time costs.
(2) Government departments can propose punitive measures for the failure to establish remote consultations, such as publicly announcing and releasing information on the results of the treatment of patient complaints against hospitals. Notably, THs exhibit greater sensitivity to low-intensity reputation damage than PHs, while the influence of medium- to high-intensity reputation damage on the final decision-making process remains inconclusive for both parties. Therefore, it is imperative to establish a clear and reasonable framework for the punishment of NERS. In addition, it is essential to tailor punitive measures and their content to the unique characteristics of hospitals at different levels. This approach ensures that the punishment measures are targeted and avoids adopting a generic approach.
(3) Government subsidies influence the decision-making process of THs and PHs during the initial stages of evolution. From the perspective of sustainable development, the economic benefits and resource sinking utility derived from establishing a remote consultation system have greater appeal for both parties involved. Moreover, the acquisition of resource sinks can significantly enhance the selection of building strategies more effectively than can an increase in economic benefits. Therefore, while ensuring an equitable distribution of government subsidies, economic benefits, and resource sinking utility, due attention should also be given to a greater proportion of the resource sinking utility for PHs. To encourage the mobile training of human resources in medical alliances in China, doctors should be sent regularly to help PHs, the construction of hardware facilities for PHs should be strengthened to address the decrease in medical resources, adjusting medical insurance policy should be considered, patients should be guided to seek medical treatment nearby, and the number of hospital consultations should be increased. This will increase the ability of soft power and hardware facilities to increase the amount of medical resources obtained by PHs.

## Abbreviations

ERS: Both tertiary hospitals and primary hospitals opt to establish a remote consultation system
NERS: Neither tertiary hospitals nor primary hospitals do not choose to establish a remote consultation system
PHs: The primary hospitals in a medical alliance in China
THs: The tertiary hospitals in a medical alliance in China

## References

[1] Yang J, Lu S, Jin J, Zhang L. Analysis of hierarchical diagnosis and treatment based on systematic thinking. Chinese Journal of Hospital Administration. 2016; 36(01): 1–5.

[2] Feng J, Lü S, Wang Z. Evaluation of medical resource sharing and patient choice: policy assessment of medical consortium construction in China. Management World. 2022; 38(10): 144–57+73+58.

[3] Heinzelmann PJ, Lugn NE, Kvedar JC. Telemedicine in the future. Journal of telemedicine and telecare. 2005; 11(8): 384–90. doi: 10.1177/1357633x0501100802 16356311

[4] Zhou J, Liu Y, Xu M, Mei L, Ruan S, Zhang N, et al. Management of breast cancer patients during the coronavirus disease 2019 pandemic: the experience from the epicenter of China, Wuhan. Clinical Breast Cancer. 2022; 22(1): e1–e7. doi: 10.1016/j.clbc.2021.04.014 34078565 PMC8099546

[5] Bhaskar S, Nurtazina A, Mittoo S, Banach M, Weissert R. Editorial: Telemedicine During and Beyond COVID-19. Frontiers in public health. 2021; 9: 662617. doi: 10.3389/fpubh.2021.662617 33796502 PMC8007781

[6] Cormi C, Ohannessian R, Sanchez S. Motivations of French physicians to perform teleconsultations during COVID-19: A mixed-method study. Telemedicine and e-Health. 2021; 27(11): 1299–304. doi: 10.1089/tmj.2020.0524 33560152

[7] Gkrouzman E, Wu DD, Jethwa H, Abraham S. Telemedicine in rheumatology at the advent of the COVID-19 pandemic. HSS Journal^®^. 2020; 16(1_suppl): 108–11. doi: 10.1007/s11420-020-09810-3 33041724 PMC7537960

[8] Du T, Li J, Li N. Empirical study on the willingness to use remote consultation among doctoes in Yan’an medical alliance based on the extended UTAUT model. Medicine and Society. 2023; 36(03): 131–7. doi: 10.13723/j.yxysh.2023.03.024

[9] Hoque R, Sorwar G. Understanding factors influencing the adoption of mHealth by the elderly: An extension of the UTAUT model. International journal of medical informatics. 2017; 101: 75–84. doi: 10.1016/j.ijmedinf.2017.02.002 28347450

[10] Wang Y-C, Ganzorig B, Wu C-C, Iqbal U, Khan HAA, Hsieh W-S, et al. Patient satisfaction with dermatology teleconsultation by using MedX. Computer Methods and Programs in Biomedicine. 2018; 167: 37–42. doi: 10.1016/j.cmpb.2018.10.015 30501858

[11] Yang H, Lü C. Ten years of “New Medical Reform”: The regional differences, dynamic evolution and influencing factors of China’s medical and health service efficiency. Chinese Journal of Management Science. 2023; 31(02): 162–72.

[12] Zhai Y, Lu W, Sun D, Zhao J. A tripartite game among government, hospitals and patients under the telemedicine background. Chinese Health Economics. 2018; 37(07): 54–7. doi: 10.7664/CHE20180714

[13] Gu H, Wu D, Han G, Xu B, Sun J. Sduty on the construction of regional remote consultation service platform in China. Chinese Journal of Health Policy. 2019; 12(07): 65–9.

[14] Xie L, Yang H, Wu Y. Medical and health resources, mortality rate of COVID-19 and optimal allocation of resource. Research on Economics and Management. 2020; 41(08): 14–28. doi: 10.3969/j.issn.1674-2982.2019.07.010

[15] Zhang L. Study on the equilibrium and optimization of medical resource allocation in metropolitan area: A case study of Shanghai. Nanjing Journal of Social Sciences. 2019; (02): 65–72.

[16] Zou L, Chen X, Xu C, Xing L, Xie Y. Design and preliminary experience of a tele-radiotherapy system for a medical alliance in China. Telemedicine and e-Health. 2020; 26(2): 235–43. doi: 10.1089/tmj.2018.0323 30892144

[17] Li Z-P, Wang J-J, Chang A-C, Shi J. Capacity reallocation via sinking high-quality resource in a hierarchical healthcare system. Annals of Operations Research. 2021; 300: 97–135. doi: 10.1007/s10479-020-03853-9

[18] Tao C, Chen X, Zheng W, Zhang Z, Tao R, Deng R, et al. How to promote the hierarchical diagnosis and treatment system: A tripartite evolutionary game theory perspective. Frontiers in Psychology. 2022; 13: 1081562. doi: 10.3389/fpsyg.2022.1081562 36687941 PMC9849701

[19] Li Z, Wang J. Hing-quality service capacity reallocation decision and profit-sharing coordination scheme design in the hierarchical healthcare systems. Chinese Journal of Management Science. 2023; 31(04): 205–17.

[20] Li J, Yang F, Mao Z. Analysis of fairness and efficiency of medical and health resource allocation in county-level of Hubei province. Statistics & Decision. 2017;(13): 114–17.

[21] Tao C, Guo T, Xu K. Measure of equalization level of medical and health resource allocation in China. Statistics & Decision. 2019; 35(24): 42–6.

[22] Wang J, Jia W. Resoures allocation and utilization efficiency in China’s healthcare sector. Finance & Trade Economics. 2021; 42(02): 20–35.

[23] Zhang L. A sduty on the effective strategies of urban hierarchical medical system in the post-epidemic era. Nanjing Journal of Social Sciences. 2020; (04): 7–13.

[24] Wu Q, Miao R, Song Y, Cheng Y, Jiang Z. Medical resource allocation contributing to hierarchical diagnosis. Industrial Engineering and Management. 2018; 23(03): 150–6.

[25] Lian W, Xue T, Lu Y, Wang M, Deng W. Research on hierarchical data fusion of intelligent medical monitoring. IEEE Access. 2019; 8: 38355–367. doi: 10.1109/ACCESS.2019.2958854

[26] Smith J M, Price G R. The logic of animal conflict. Nature, 1973;246(5427): 15–8. doi: 10.1038/246015a0

[27] Wang X, Du R, Ai S, Zhang Z. An evolutionary analysis of community hospital and patient behavioral choice in the context of telemedicine. Industrial Engineering and Management, 2015; 20 (02): 130–7.

[28] Yao X, Wen Z, Lu Y, Zheng R. Evolutionary Game Study on the Promotion Strategy of Telemedicine in the Gontext of Gounty Medical Comunity. Price: Theory & Practicev, 2023; (03): 152–5+206.

[29] Fan Y, Liu S, Liu J, Javed SA, Fang Z. Habit or utility: A key choice point in promoting the adoption of telehealth in China. Complexity, 2020; 2020: 1–11. doi: 10.1155/2020/5063756

[30] Sun X, Zhou W, Feng Y. Mobile healthcare platforms’ sustainability: the perspective of health information quality. Frontiers in Public Health, 2023; 10: 1059252. doi: 10.3389/fpubh.2022.1059252 36685000 PMC9853185

[31] Zhu G, Liu H, Feng M. An evolutionary game-theoretic approach for assessing privacy protection in mHealth systems. International journal of environmental research and public health, 2018; 15(10): 2196. doi: 10.3390/ijerph15102196 30297659 PMC6210030

[32] Shen S, Zhang B. Hierarchical diagnosis and treatment, primary diagnosis and treatment, and construction of primary medical and health institutions. Academia Bimestris. 2016; (02): 48–57.

[33] Mu J, Sun Z, Zhao J, Zhai Y. Analysis about the value of telemedicine based on practice in Henan provincial telemedicine center. Chinese Health Economics. 2014; 33(10): 15–7. doi: 10.7664/CHE20141004

[34] Cai Y, Zhai Y, Hou H, Zhao J. Cost-effectiveness analysis based on of telemedicine network roles. Chinese Health Economics. 2014; 33(10): 8–10. doi: 10.7664/CHE20141002

[35] Yu J, Zhang T, Liu Z, Hatab AA, Lan J. Tripartite data analysis for optimizing telemedicine operations, Evidence from Guizhou province in China. International journal of environmental research and public health. 2020; 17(1): 375. doi: 10.3390/ijerph17010375 31935950 PMC6981610

[36] Gao L, Wang X. Research on pricing srtategy and coordination of medical service supply chain based on patient choice. Chinese Journal of Management. 2020; 17(03): 422–30. doi: 10.3969/i.issn.1672-884x.2020.03.012

[37] Pang R, Li S. Structural imbalance of medical resource allocation and “kanbing gui” in China: From the perspective of referral system. Modern Economic Science. 2022; 44(03): 97–110. doi: 10.1258/135763305775013554

[38] Andersen AR, Nielsen BF, Reinhardt LB. Optimization of hospital ward resources with patient relocation using Markov chain modeling. European Journal of Operational Research. 2017; 260(3): 1152–63. doi: 10.1016/j.ejor.2017.01.026

[39] Selten R, Selten R. A note on evolutionarily stable strategies in asymmetric animal conflicts. Springer. 1988. 10.1016/s0022-5193(80)81038-1 7412323

[40] Ritzberger K, Weibull JW. Evolutionary selection in normal-form games. Econometrica: Journal of the Econometric Society. 1995: 1371–99. doi: 10.2307/2171774

[41] Friedman D. On economic applications of evolutionary game theory. Journal of evolutionary economics. 1998; 8: 15–43. doi: 10.1007/s001910050054

[42] Cai Y, Zhang X, Shi J, et al. Evolution Analysis of Doctors and Patients’Behavior Choice Under the Background of Telemedicine. Chinese Hospital Management, 2019; 39(09): 15–9. doi: 10.1258/135763305775013554

[43] Zhang L, Wang X, Xiao H, Ma C, Li X, Dai G, et al. Governance mechanisms for chronic disease diagnosis and treatment systems in the post-pandemic era. Frontiers in Public Health, 2022; 10: 1023022. doi: 10.3389/fpubh.2022.1023022 36582374 PMC9792788

[44] Cai L, Shi J, Li L, Shan E. Mechanism of benefit distribution in medical treatment combination based on the principle of effective distribution of graph- restricted cooperative game. Journal of Systems & Management. 2021; 30(02): 393–400.

